# Translating primary care to telehealth: analysis of in-person paediatric consultations and role of carers

**DOI:** 10.3399/BJGPO.2024.0030

**Published:** 2025-02-26

**Authors:** Simon Chan, Tasneem Khandaker, Yifu Li, Tim M Jackson, Hania Rahimi-Ardabili, Annie YS Lau

**Affiliations:** 1 Centre for Health Informatics, Australian Institute of Health Innovation, Macquarie University, Sydney, Australia

**Keywords:** child health, telehealth, telemedicine, general practice, primary healthcare

## Abstract

**Background:**

The usage of telehealth in general practice has risen substantially since the COVID-19 pandemic. Over this time frame, telehealth has provided care for all patient demographics, including the paediatric population (aged ≤18 years). However, the translatability of telehealth (that is, whether in-person tasks can be supported remotely via telehealth) rarely considers the paediatric population or their carers.

**Aim:**

To examine the degree of translatability to telehealth for in-person GP consultations on paediatric patients with consideration of the carers’ roles.

**Design & setting:**

This study screened 281 videos of in-person GP consultations set within UK general practices, and 20 of them were eligible for analysis.

**Method:**

A secondary analysis of in-person GP consultations was undertaken to examine tasks, physical artefacts, examinations, and the interaction between carer, patient, and GP. A novel scoring method revolving around two key metrics, taking into consideration the carer, was designed to analyse whether the tasks performed can be supported via telehealth.

**Results:**

Analysis of 20 eligible consultations revealed 13 distinct physical examinations, 19 physical artefacts, and 17 clinical tasks. Of these 17 clinical tasks, 41% were deemed ‘easily translatable to telehealth,’ 29% 'moderately translatable with patient-provided equipment', 12% 'potentially translatable', and 18% 'currently untranslatable'. The average telehealth translatability score was 6.1/10, which suggests possible challenges with telehealth support. Regarding carer involvement, 90% of consultations involved collecting patient history, 70% placation of child, and 40% had physical support during examinations.

**Conclusion:**

Tasks performed during paediatric in-person GP consultations may not be easily translatable to telehealth and caution should be exercised when considering their translatability to telehealth.

## How this fits in

Amid the rise in telehealth during the COVID-19 pandemic, researchers have extensively studied the effectiveness of telehealth among healthcare professionals. Existing literature indicates successful management of paediatric conditions by health practitioners through telehealth. Nonetheless, limited research within a primary care context has explored the translatability of tasks typically conducted by general practitioners (GPs) in face-to-face paediatric consultations to the telehealth medium. This research assesses the translatability of clinical tasks performed for this specific patient demographic.

## Introduction

GP consultations underpin the foundation of patient care as they are the primary point of healthcare access for the majority of patients. While they are traditionally conducted in person, the COVID-19 pandemic prompted a significant surge in telehealth consultations worldwide, with more than 95 000 practitioners delivering 118.2 million telehealth consults to 18 million Australian patients between March 2020 and July 2022.^1^ Telehealth encompasses the remote delivery of healthcare services and communication between patients and healthcare providers, and includes not just phone calls but also consultations via video, texts, and mobile apps. Telehealth has become increasingly acceptable in general practice; for example, 84% of patients in Adelaide Hills in South Australia have expressed interest in continuing telehealth consults even outside the pandemic lockdown periods.^2^

The rapid shift to telehealth raises concerns about the adaptability and readiness of health systems to transition effectively, especially in supporting tasks typical of in-person consultations such as physical examinations.^1,2^ The critical question remains: under what circumstances and for which patient groups is teleconsultation an appropriate mode for in-person consultations in general practice?

A substantial proportion of the Australian paediatric population visits a general practice, with 79% of children aged <15 years seeing a GP within a 12-month period.^3^ The conditions these patients present with vary, encompassing acute issues, such as upper respiratory tract infections, as well as chronic illnesses such as asthma and depressive disorders.^4^ Throughout the COVID-19 pandemic, such conditions still required regular GP care, and these patients were often accompanied by a carer (for example, parent). Consequently, it is crucial to assess the suitability of incorporating telehealth as a supplement to in-person consultations for this specific age group (aged ≤18 years).

The carer’s role in paediatric consults is critical as up to 90% of consults are led by the carer (for example, parent) without substantial participation from the child.^5^ The carer assists the consultation by supplying the child’s history, helps placate the child physically, and provides emotional support during the consultation.^6,7^ Thus, it is essential to evaluate the carer’s role during GP paediatric in-person consultations, and explore ways to support both the child and the carer during teleconsultation.

Telehealth has improved the provision of health services in general and several studies have showcased its effectiveness in primary care.^8,9^ White *et al*’s study highlighted that effective communication within teleconsultations is important to achieve optimal patient satisfaction and clinical outcomes.^10^ Emerging evidence supports telehealth’s effectiveness for paediatric care, where patients, healthcare providers, and carers may all benefit from using a hybrid in-person and telehealth approach.^11,12^

Few studies have explored the physical tasks performed and the role of carers during in-person paediatric GP consultations.^5,10–12^ Additionally, there is a need to examine the possibility of translating these in-person tasks during paediatric GP consultations to telehealth, as well as how carers can be supported. This study addresses these gaps, defining translatability as the ability of in-person tasks to be performed remotely over telehealth. Focusing on in-person GP consultations with paediatric patients and carers, this study aims to provide insights into GP–patient–carer interactions, examinations, and physical tasks. The analysis will determine the extent to which these paediatric GP consultations can be translated to telehealth.

## Method

### Study design

The data collection and analysis process involved secondary analysis of video recordings of GP consultations obtained in a 2017 NHS project called *Harnessing resources from the internet to maximise outcomes for GP consultations (HaRI): a mixed qualitative methods study to investigate the internet use in GP.* ^13^ This HaRI study contained 281 video recordings and associated transcripts for consultations obtained from 10 GPs working across the UK at eight different clinics.

### Data collection

Two researchers (SC, TK) independently screened the initial pool of 281 GP consultation transcripts based on the study eligibility criteria to include (i) patients aged ≤18 years , with a primary focus on the paediatric patient in the presentation. Transcripts were included when (ii) the patient was examined and/or (iii) there was the use of a physical artefact(s) (see supplementary Table S1 for detailed eligibility criteria). There were 36 suitable paediatric GP consultations. However, 16 consultations lacked consent for video and transcript analysis, had non-deidentified data, or lacked physical artefact and/or examination use. Consequently, 20 eligible consultations remained for final analysis (see supplementary Figure S1).

### Data analysis

The data analysis involved five main categories.

#### Descriptive analysis

Both researchers (SC, TK) independently reviewed eligible video recordings, extracting relevant patient and consultation characteristics. Descriptive statistical analysis was then applied to identify trends.

#### Time analysis

One researcher (SC) extracted the total consultation length, time spent on physical examinations, and number of physical examinations in each consultation from the eligible video recordings.

#### Video and transcript analysis

Data from eligible paediatric consultations, physical examinations, artefacts, and clinical tasks were extracted and recorded in an Excel spreadsheet. The following seven categories were established: (1) presence of physical examination; (2) type of physical examination(s) conducted, for example, temperature check; (3) presence of a physical artefact; (4) type of physical artefact(s) utilised, for example, thermometer; (5) accessibility of the physical artefact used (three subcategories: readily found in patient’s home, easily acquired through provision, or not easily acquired by the patient); (6) clinical task(s) during the paediatric consultation, for example, medication discussion; and (7) involvement of carer in physical examination, task, or both. Subsequent analysis was performed on the extracted data. We considered factors such as cost, prevalence in households, and variations in socioeconomic status when determining how accessible the physical artefacts used during the consultations are.

#### Data verification

This process involved a third researcher (RL), independently re-analysing all eligible video recordings and transcripts to validate the initial data extraction. Any discrepancies related to physical examinations, artefacts, and clinical tasks were cross-checked against the initial analysis and adjusted.

#### Translatability to telehealth analysis

**Figure 1.**
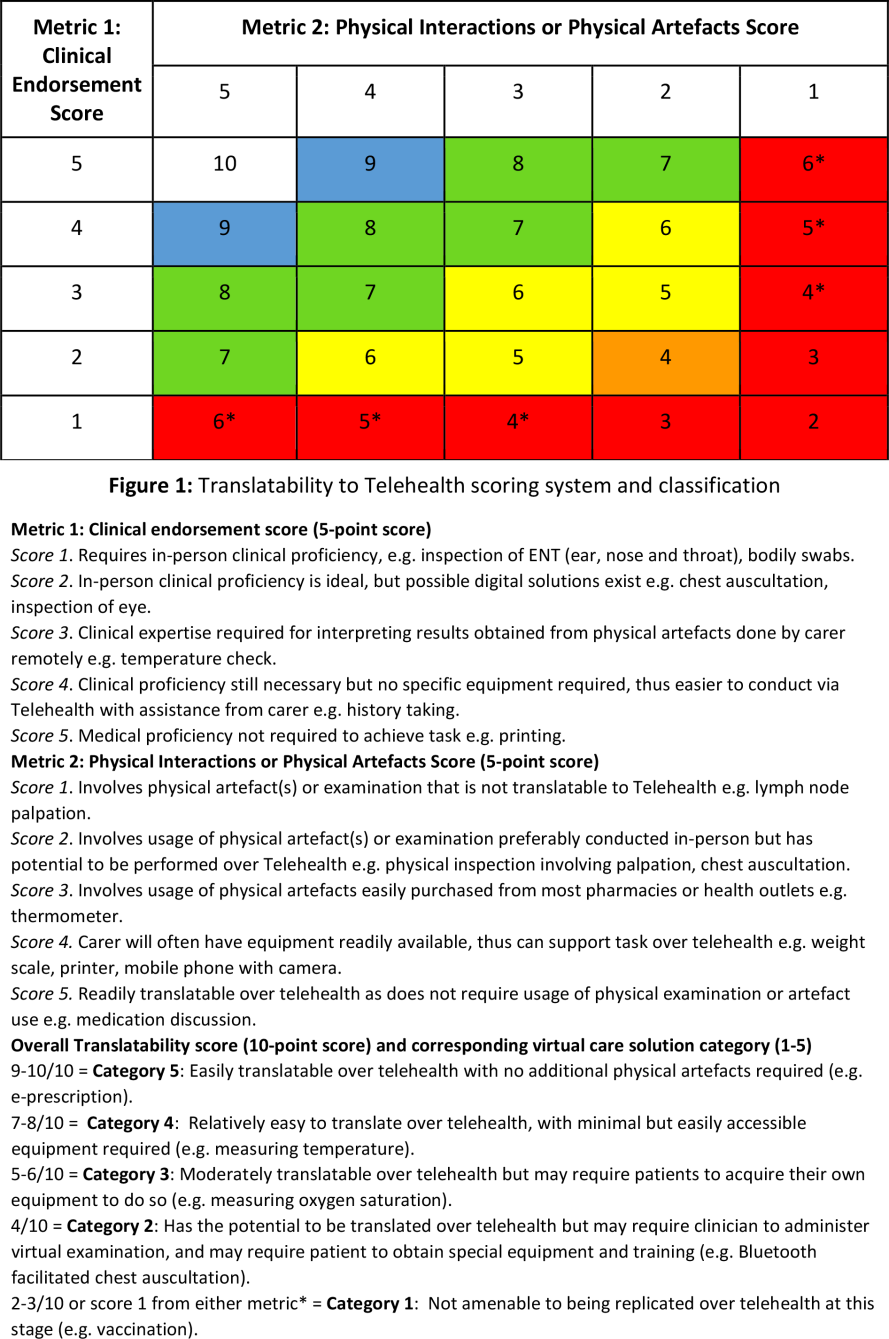
Translatability to telehealth scoring system and classification

The translatability of in-person clinical tasks for eligible paediatric GP consultations was assessed using a scoring system. Adapted for paediatric tasks and carer consideration, this system is based on three previous studies and uses a four-step approach.^14–16^ The first step is to evaluate the extent to which a clinical task requires ‘clinical expertise’ using a 5-point scoring system. The second step is to evaluate the extent to which the clinical task requires ‘physical interaction and/or physical artefacts’ with another 5-point scoring system. The third step combines steps 1 and 2 for a total score out of 10. This score was then plotted on the matrix in Figure 1 to determine translatability to telehealth. The complete scoring system process can be found in Figure 1. The fourth and final step is to assign each task to a telehealth solution category. This step was developed in the original research studies.^14–16^ This depends on the carer’s support level, as these tasks often exceed the paediatric patient’s abilities and require assistance from the parent or guardian. The telehealth solution considers both the translatability of the clinical task and the necessary support for the carer (detailed examples in Table 1).

**Table 1.** Telehealth solution category examples

Translatability to Telehealth score	Example
Category 5	Doctor, carer and patient can effortlessly exchange information via video or telephone, for example, medication discussion, electronic prescription
Category 4	Carer conducts physical assessment on behalf of doctor at home and then reports relevant findings, for example, measurement of temperature
Category 3	Carer acquires required artefact(s) then performs and reports findings. Virtual instructions or guidance may be necessary, for example, measurement of oxygen saturation
Category 2	Doctor performs virtual examination. Patient needs specialised equipment or training, for example, Bluetooth-facilitated chest auscultation
Category 1	Not suitable for telehealth

**Table 2.** Clinical tasks, physical artefacts, and translatability scores

Clinical task	Physical artefact(s)	Role of carer	Clinical score	Artefact score	Translatability score	Solution category
**Physical examinations**						
Temperature check	Thermometer	Physical support	3	3	6/10	3
Inspection of skin	Nil	Placating the child	2	4	6/10	3
Chest auscultation	Stethoscope	Demonstration of technique	2	2	4/10	2
Inspection of ENT	Otoscope, tongue depressor	Physical support	1	1	2/10	1
Physical inspection involving palpation	Nil	Physical support, placating the child	2	2	4/10	2
Inspection of eye	Source of light, for example, medical torch, phone	Physical support	2	3	5/10	3
Weight measurement	Weight scale	Placating the child	3	4	7/10	4
Lymph node palpation	Nil	Physical support	1	5	6/10	1
Measuring oxygen saturation	Pulse oximeter	Placating the child	3	3	6/10	3
Swab of ear	Ear swab, specimen tube	Physical support	1	3	4/10	1
Smell of breath	Nil	Demonstration of technique	3	4	7/10	4
Scalp sample obtainment	Hairbrush, specimen tube	Physical support, placating the child	2	3	5/10	3
**Management**						
Discussions about medications	Physical medication, for example, tablet, puffer	Interpretation of instructions	4	5	9/10	5
Writing pharmaceutical prescriptions	Computer, printer, prescription	Interpretation of instructions	4	4	8/10	4
Pathology test(s) discussion	Printer, electronic or physical results	Interpretation of instructions	4	4	8/10	4
**Investigations**						
Generating specialist or allied health referral letters	Referral letter, printer	Interpretation of instructions	4	4	8/10	4
Generating pathology request	Computer, printer, request form	Interpretation of instructions	4	4	8/10	4

ENT = ear, nose, and throat

## Results

### Patient and consultation characteristics

In total, 20 paediatric GP consultations were analysed in this study. Most patients were male (70%) and aged 0–6 years (65%). A carer supported clinical tasks in 60% of cases. See Supplementary Table S2 for detailed patient and consultation characteristics.

### Physical examinations in GP paediatric consultations

**Figure 2.**
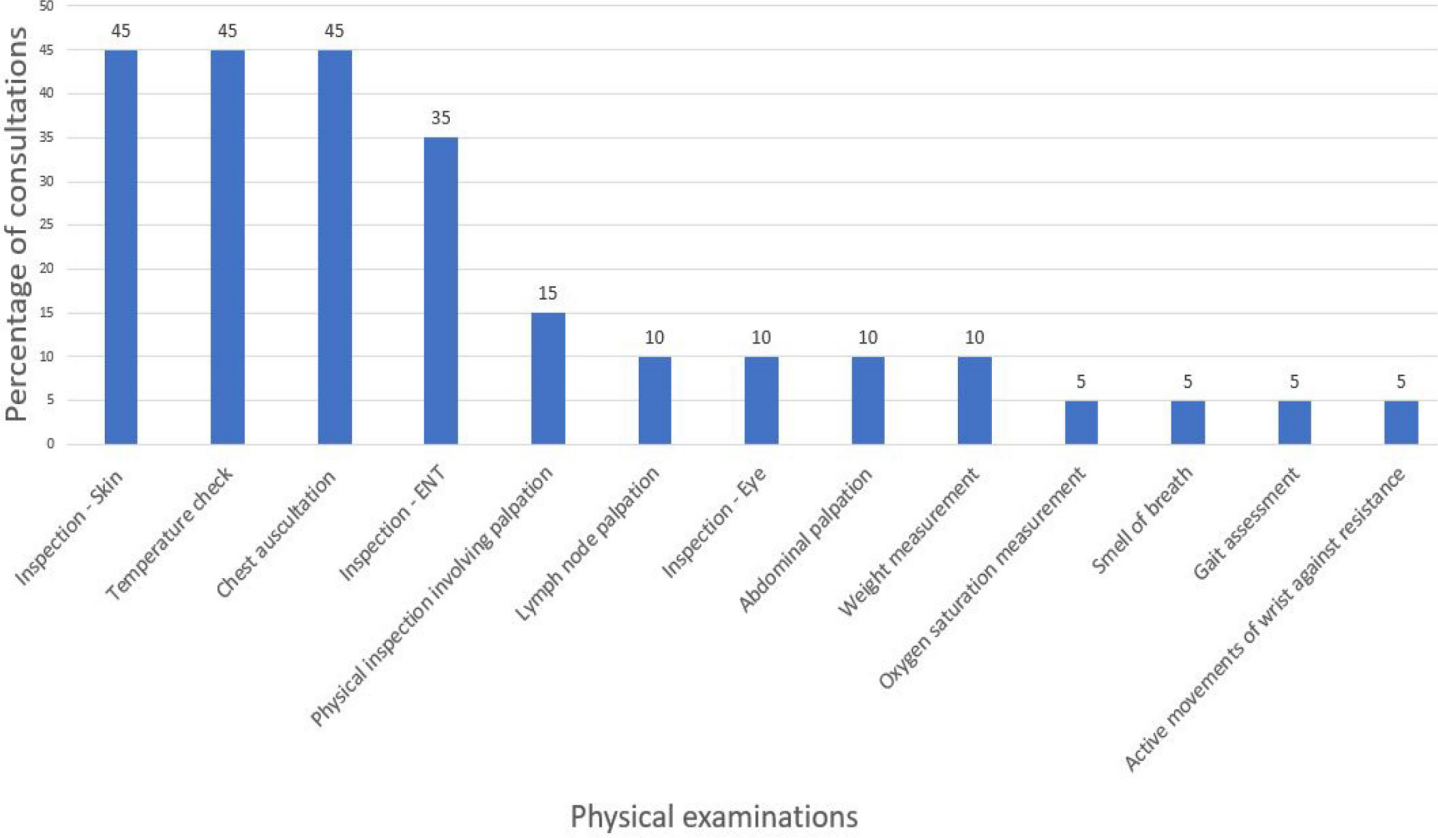
Frequency of physical examinations performed during in-person paediatric consultations, as a percentage of total consultations (*n* = 20). ENT = ear, nose, and throat.

Figure 2 below demonstrates the frequency of physical examinations across the 20 consultations. From these 20 consultations, 13 different physical exams were performed. Ninety-five per cent (*n* = 19/20) of the consultations contained at least one physical examination.

### Time analysis of physical examination(s)

There was a total of 44 physical examinations performed across the 20 paediatric consultations (refer to Supplementary Table S3). Analysis revealed the average length of the consultations was 9 minutes 29 seconds, with an average time spent on physical examination of 1 minute 47 seconds. It was also determined that, on average, 21% of total consultation time was spent on physical examination(s).

### Physical artefacts utilised during in-person paediatric consultations

**Figure 3.**
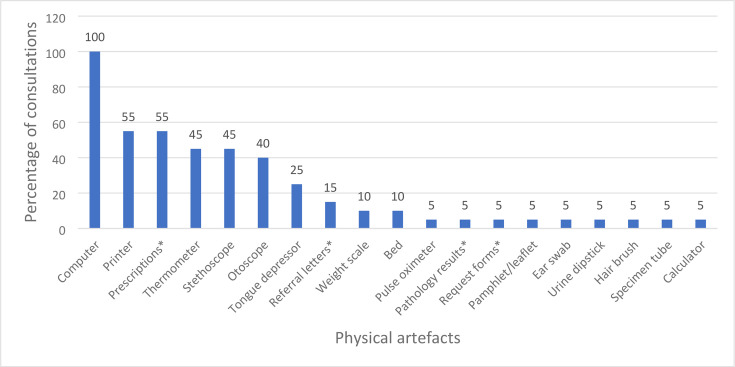
Frequency of physical artefacts utilised during in-person paediatric consultations, as a percentage of total consultations (*n* = 20). ^a^These physical artefacts were dispensed either by GP or at reception desk

Figure 3 illustrates the frequency of physical artefacts observed throughout the 20 consultations. In summary, 19 physical artefacts were identified across the consultations and from this, 11 were identified as readily available in the carer or patient’s home, six were identified as easily acquired through purchase, and two were defined as not easily obtained (See supplementary Tables S4–S6 for visual examples).

### Clinical tasks performed during in-person paediatric consultations

**Figure 4.**
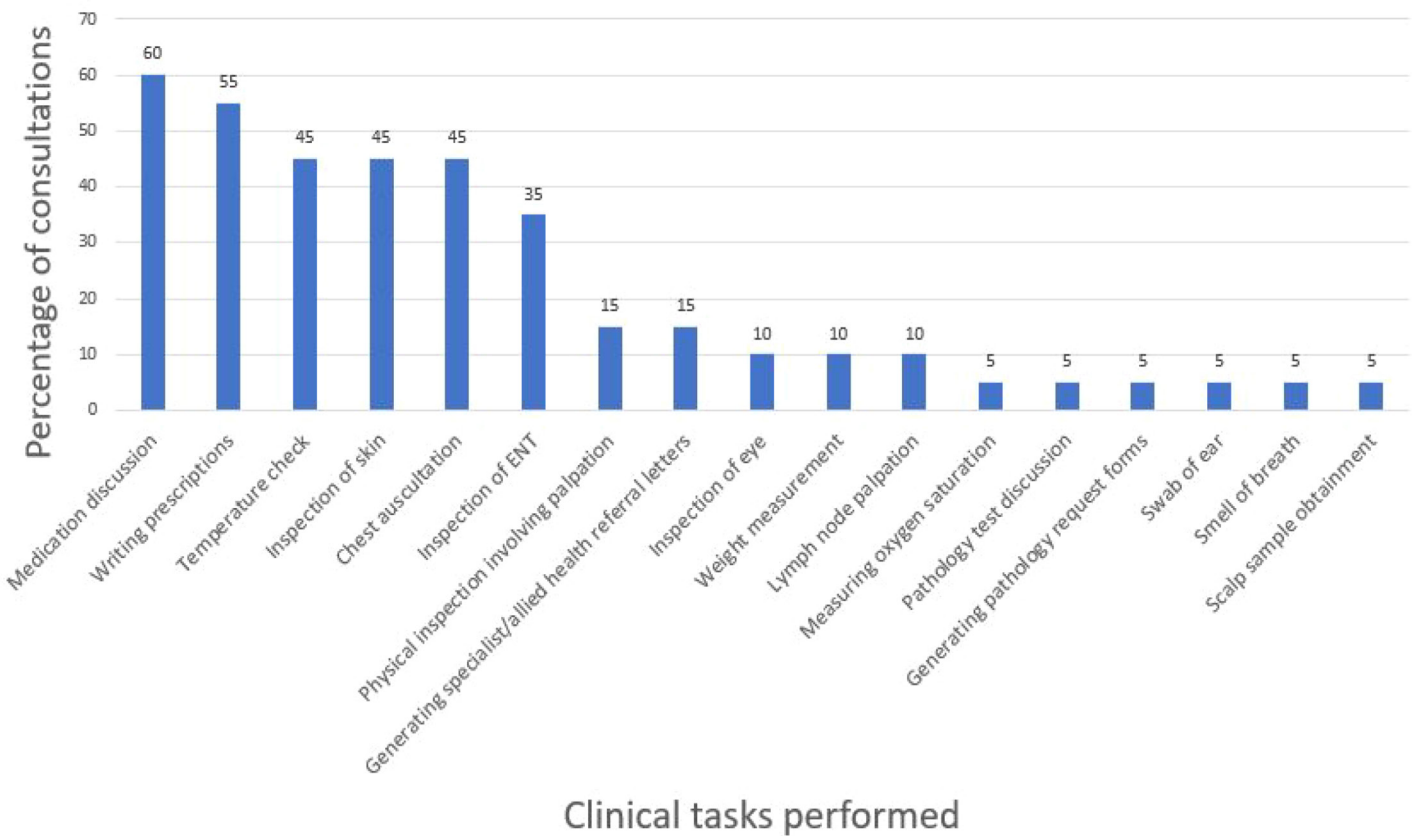
Frequency of clinical tasks performed during in-person paediatric consultations, as a percentage of total consultations (*n* = 20). ENT = ear, nose, and throat.

Figure 4 illustrates the frequency of clinical tasks observed throughout the 20 consultations. Overall, there were 17 tasks noted across the 20 consultations. From the 17 tasks, 71% (*n* = 12/17) involved physical examination, and 65% (*n* = 11/17) of these tasks required physical artefacts at least some or all of the time.

### Clinical tasks translatability to telehealth

The translatability of 17 clinical tasks, the artefacts used, and the role of the carer can be found in Table 2. The average scores for these tasks were as follows: 2.6/5 for clinical endorsement; 3.2/5 for physical interactions or physical artefacts; and 6.1/10 for translatability to telehealth. Telehealth solution categories were assigned to each task:

- 18% (*n* = 3/17) were categorised as category 1 (unable to be reproduced over telehealth at this point in time): lymph node palpation, inspection of ear, nose, throat (ENT), and swab of ear.
- 12% (*n* = 2/17) were categorised as category 2 (potentially translatable to telehealth but may require a doctor to perform virtual examination, or patient needs special equipment or training): chest auscultation, and physical inspection involving palpation.
- 29% (*n* = 5/17) were categorised as category 3 (moderately translatable to telehealth but may require patient-provided equipment): temperature check, inspection of skin, inspection of eye, measuring oxygen saturation, and scalp sample obtainment.
- 35% (*n* = 6/17) were categorised as category 4 (relatively easily translatable over telehealth as requires only readily available physical artefacts): writing pharmaceutical prescriptions, weight measurement, smell of breath, pathology test(s) discussion, generating specialist or allied health referral letters, and pathology request forms.
- 6% (*n* = 1/17) were categorised as category 5 (easily translatable over telehealth with no physical artefacts required): medication discussion.

### Role of carers

In 90% (*n* = 18/20) of consultations, carers interpreted GP instructions, providing medical history and taking further action. Placation of the child through physical (for example, patting) and verbal cues (for example, asking questions) occurred in 70% (*n* = 14/20), while social cues (for example, smiling) were evident in 40% (8/20). Physical support, involving holding the child and undressing or dressing, was observed in 40% (*n* = 8/20) of consultations (See Table S7 for visual examples). Maintaining stillness of the child is often crucial for accurate assessment and for the 40% of consultations where a carer was required, this is to ensure the safety and effectiveness of the physical examination. Calming the child and demonstrating techniques were seen in 15% (*n* = 3/20) and 5% (1/20) of consultations, respectively. Supplementary Table S8 details the carers' roles in paediatric GP consultations, showcasing tasks, modes, and associated frequencies, as well as various physical, verbal, and social cues exhibited by carers towards paediatric patients.

## Discussion

### Summary

The study’s average translatability to telehealth score was 6.1 out of 10. This score suggests that primary care clinical tasks for the paediatric cohort may not be easily translatable to telehealth and thus caution is advised in adopting GP teleconsultations with paediatric patients. Results revealed that 95% (*n* = 19/20) of consultations involved at least one of the 13 distinct physical examinations, with a majority requiring physical artefacts that are readily available at the patients’ home.

However, key tasks such as ENT inspection and chest auscultation were deemed not readily translatable to telehealth. Classifying tasks with their respective telehealth solution category has highlighted existing gaps in telehealth, emphasising the need for further research on improving patients' and carers' support during telehealth, and eliminating barriers to the use of telehealth.^11,12,17^

The role of the carer is crucial during paediatric consultations where they contribute significantly from placating the child to providing physical and emotional support during physical examination. Physical and verbal cues to calm the patient were present in 70% (*n* = 14/20) of consultations. Older children required less carer support. Further research is needed to understand how paediatric patients and carers are being supported in virtual care, to identify unmet needs, and to develop skills for safe and effective participation in virtual care by all parties, including clinicians, paediatric patients, and carers.

### Strengths and limitations

This study’s main strength lies in analysing unique data from the HaRI database, a primary source of in-person primary care consultations. The objective examination of data via video and transcript analysis minimises biases (for example, recall and confirmation) and measurement errors inherent in self-reported methods such as questionnaires. Implicit bias was mitigated by analysing multiple GP consultations and multiple coders, while video analysis allowed for a deeper exploration of carer roles.

However, limitations include a small sample size (20 consultations), which potentially means the findings are not indicative of the entire GP paediatric patient cohort. Despite this, our study did encapsulate a majority of the more common reasons for a paediatric GP visit, with the exception of allergies and headaches.^18^ Findings from this study may not be representative and applicable to other settings such as non-English speaking countries or those with different healthcare systems.

Furthermore, patient factors impacting telehealth effectiveness, such as digital literacy, language barriers, and socioeconomic status, were not considered in this study. Lastly, the study does not contain consultations with certain paediatric populations such as those with physical or intellectual disabilities. This highlights that this study is not fully representative of the entire paediatric population; future studies are needed to explore this avenue.

### Comparison with existing literature

Our team has used the same approach and dataset to assess telehealth translatability of in-person GP consultations for respiratory,^14^ diabetes and cardiovascular (CVD),^15^ and chronic conditions.^16^ These studies, which employed the same rating system as the current study, rated the telehealth transferability as moderate (7.2/10 for diabetes and CVD,^15^ 6.7/10 for respiratory,^14^ and 7.0/10 for chronic conditions).^16^ Other studies analysing telehealth usage for paediatric care concluded that telehealth was comparable with in-person services, emphasising its positive impact.^11,19^ The involvement of carers in placating children through physical and verbal cues, observed in this study, aligns with the University of Michigan findings.^20^ Furthermore, various studies highlighted significant carer involvement in decision making during consultations, consistent with our study.^5,20^

Clinical tasks such as chest auscultations categorised as ‘potentially translatable to telehealth’ is consistent with emerging digital solutions, where several papers provided guidance on performing digital chest auscultations via Bluetooth-connected stethoscopes and also musculoskeletal examinations.^21,22^ Although ‘ENT inspection’ is currently deemed ‘unable to be reproduced over telehealth’, evidence supports the use of digital otoscopes for ear examinations and smartphone videos for oropharyngeal examinations.^23^ However, availability of these digital tools for GPs and patients during teleconsultation remains unclear.

### Implications for research and practice

Clinical tasks in paediatric primary care often require specialised instruments and clinical knowledge, posing safety concerns for accurate diagnosis via remote examination.^24^ The involvement of both carer and child in consultations raises the possibility of medical errors owing to the inability to assess accuracy of home-based equipments, as well as the lack of expertise among children and carers in conducting patient (or self-)examination. Research is needed to alleviate safety concerns that arise from telehealth examination.

Currently, certain clinical tasks, such as ENT inspection, are challenging to reproduce over telehealth, emphasising the carer’s significant role in facilitating these tasks when new technologies emerge. Further research should analyse viability of existing digital solutions and equip carers with necessary skills for aiding with physical assessments during telehealth.

While telehealth has efficiently triaged patients and increased access to care during the pandemic, it has accentuated disparities for vulnerable populations, such as those on a low income or with low digital literacy.^12,24,25^ To maximise telehealth’s potential, research should focus on reducing social and technological barriers for vulnerable paediatric patients and their carers.

Long-term virtual care may lead to ‘low-value care’, which is when care provided is ineffective, unwanted, or inefficient.^26^ Thus, this emphasises the need to understand under what circumstances telehealth is appropriate, safe, and acceptable to ensure high-value care is delivered and that valuable resources are not wasted.

The 'translatability to telehealth' scoring system holds potential for refining telehealth use, aiding health systems to decide whether a clinical scenario is appropriate for telehealth. Ultimately, emerging evidence supports that telehealth could be effective for paediatric care under the right circumstances, suggesting that a hybrid in-person and telehealth approach could benefit patients, healthcare providers, and carers.^11,12^

In conclusion, while some of the tasks in paediatric GP consultations were identified as translatable to telehealth, the overall translatability score remained low. Specialised instruments and carer’s support are frequently necessary in paediatric primary care, raising questions on how to achieve appropriate, safe, and acceptable remote consultations for paediatric patients. Further research is required to determine the optimal combination of in-person and telehealth involvement for paediatric patients and their carers.

## Data

The dataset relied on in this article is available from the corresponding author on reasonable request subject to ethical approval.
